# Spatio-Temporal Patterns of Land Use and Cover Change from 1990 to 2010: A Case Study of Jiangsu Province, China

**DOI:** 10.3390/ijerph16060907

**Published:** 2019-03-13

**Authors:** Ge Shi, Peng Ye, Liang Ding, Agustin Quinones, Yang Li, Nan Jiang

**Affiliations:** 1 Jiangsu Center for Collaborative Innovation in Geographical Information Resource Development and Application, Nanjing Normal University, Nanjing 210023, China; 161301020@stu.njnu.edu.cn (G.S.); 161301027@stu.njnu.edu.cn (P.Y.); li.yang@njnu.edu.cn (Y.L.); 2 Key Laboratory of Virtual Geographic Environment, Ministry of Education, Nanjing Normal University, Nanjing 210046, China; 3 School of Geographic Science, Nanjing Normal University, Nanjing 210046, China; 4 Lyles School of Civil Engineering, Purdue University, 550 Stadium Mall, West Lafayette, IN 47907, USA; quinona@purdue.edu; 5 College of Computer and Information, Hohai University, Nanjing 210098, China; 170207040003@hhu.edu.cn; 6 Information Center of Jiangsu Natural Resources Department, Nanjing 210017, China

**Keywords:** urbanization, land use and cover change, Markov, logistic regression model, driving forces, Jiangsu province

## Abstract

Land use and cover change (LUCC) is one of the most significant parts of global environmental changes, which reflects the interaction between human society and natural resources. In China, the urbanization process is experiencing a rapid sprawl since the reform and open program in 1978, and there has been a serious change in situation in the human–land relationship. In this paper, taking Jiangsu province located in the eastern coastal developed region as an example, the historic evolution process of the land use situation from 1990 to 2010 was explored. Landsat images from three periods were analyzed, using the land use transition matrix model, the land use dynamic degree model, and the land use degree model to evaluate the LUCC of Jiangsu during two research periods from 1990 to 2000 and from 2000 to 2010. Additionally, logistic regression models and some quantitative analysis were applied to identify the major potential driving factors behind the LUCC during the research period based on different dimensions. The results showed the following: (1) the most obvious change was the continuous increase of built-up area and the decrease of arable land, which reflected the deterioration of the ecological environment and the accelerate of the urbanization trend. (2) The land use change dynamic degree from 2000 to 2010 was much greater than that from 1990 to 2000. (3) Socio-economic elements and human activities were the major driving forces of LUCC in Jiangsu province. Amongst these forces, the driving factors of the population change, GDP, per capita household income, and per capita housing area have an obvious effect on the arable land loss and the built-up area expansion.

## 1. Introduction

Land use and cover change (LUCC) is one of the most significant parts of global environmental change, which is a reflection of the interaction between human society and natural resources [1,2,3]. Global environmental change is closely related to humanity, thus numerous topics have been put forward to investigate the human–land interactions, such as climatic issues, population burning, energy shortage, natural resource shortage, etc. [4,5,6,7]. Since 1992, the importance of LUCC for long-term sustainable planning has been recognized globally, when LUCC research was identified as the key project on the agenda for the 21st century as released by the United Nations. This has attracted significant attention from scholars and experts in related fields and numerous studies have already been reported around the world [5,8,9]. According to the World Bank Report, one of the most important reasons for land use change is the increasing demand for built-up area for urban expansion [10]. In high-speed developing countries, it is crucial to better manage land use planning as an imbalance between land and humanity may result in countless side effects. Thus, the evolution process of LUCC and its driving forces should be carefully studied [11,12]. 

Meanwhile, China has experienced a significant economic development period since the reform and open program in 1978, especially the eastern-developed regions like the Yangtze River Delta region. LUCC has also experienced a correspondingly huge conversion since then and in different regions across China [13]. The most obvious change is the expansion of the built-up area as part of the process of rapid urbanization, as well as the degradation of arable land and grasslands in the northern region and southern coastal regions, and water area loss and deforestation in the southern region [14,15]. Scholars in different fields have studied LUCC from the perspective of ecology, historical evolution, human geography, real estate, economy, etc. [16,17,18,19]. Some results have shown that the urban sprawl is occurring all over the world, not only in China, but they have also raised serious concerns including the human–land issues [20,21]. While China’s urbanization process is experiencing an expansion, a well-organized land use plan is crucial for future sustainable development. It is necessary to learn more about the change process of LUCC and the socio-economic driving forces in order to better manage the nationwide land use planning process [22,23,24]. 

There are numerous studies on land use and cover change in different regions across the world, focusing mainly on the land use change situation during certain period, the driving forces of LUCC, the forward result of LUCC, the planning adaption for better city management, etc. [3,25]. The above-mentioned research topics have been explored on a macro scale to learn the process and evolution of land use development. From a microscale perspective, some statistical models have been applied to forecast future trends [1,26,27,28]. Lambin used Landsat images to discover the changes of LUCC between different time periods, as well as analyzing the cause [1]. Liu, Mooney, Foley, Moran, Turner, and some other related scholars have investigated the change process of LUCC globally from rural to urban areas [8,11,15,29,30]. Lambin, Le, Ralha, et al. applied various micro models like the multi-agent system model and Conversion of Land Use and its Effects (CLUE) model to simulate the future development of LUCC [31,32,33]. Li and Cheng analyzed the current LUCC situation and the development trends in less developed regions in western China [34,35]. Zhang, Yu, Liu, and Du applied numerous statistical models to discover the land use patterns and the driving forces of changes between different regions [10,36,37,38]. The existing research on the driving forces of land use changes mainly describes the spatial and temporal patterns of the current land use situation, and then, it uses statistical models to quantify the driving forces for a certain region.

To summarize, the existing studies have used numerous statistical models to describe the evolution process of land use, and to simulate its future development trend globally. However, there is a gap in the research on LUCC and its driving forces, especially on socio-economic aspects in places experiencing rapid development. In this paper, LUCC in Jiangsu province was explored, and the evolution of land use changes were quantified for the past 20 years from a macro perspective. With the technical support of remote sensing and GIS technology, this paper analyzed the land use patterns and evolution characteristics using statistical models, which provided a deep understanding of land use, and enabled the conclusion of the corresponding driving forces behind changes in various types of urban data like internet point of interest data and statistical bureau data. The objective of this study was to investigate the spatio-temporal characteristics and potential driving forces of LUCC during the rapid urbanization period in Jiangsu province, based on Landsat image data, statistical data, and internet point of interest data. To ensure sustainable regional development, the study results aimed to provide constructive references and useful guidance for the Planning Department to optimize comprehensive urban planning. The results and conclusions from this LUCC study can also be used to study similar regions that are also experiencing rapid economic development. 

## 2. Materials and Methods 

### 2.1. Study Area

**Figure 1:**
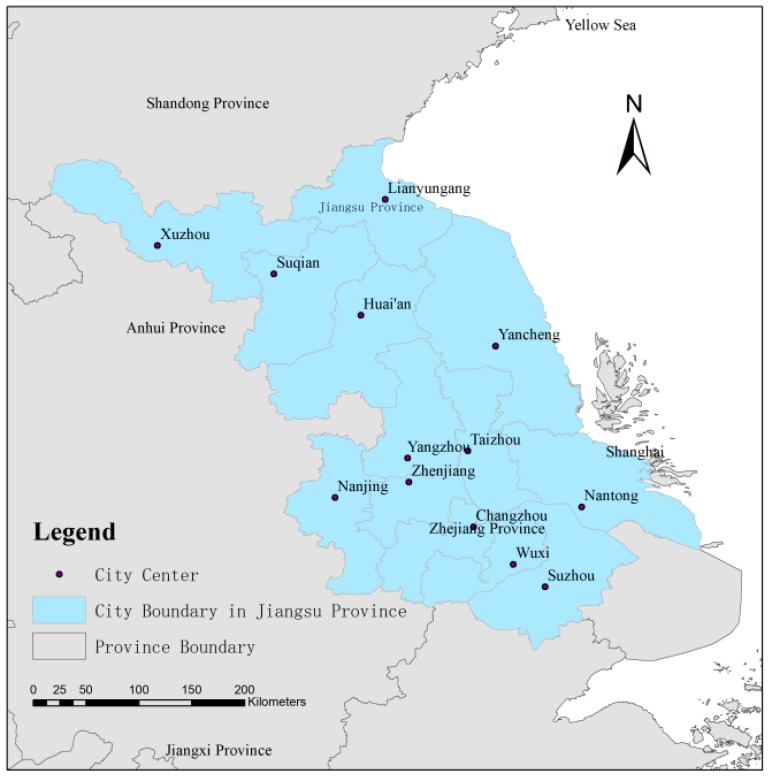
The geographical location of Jiangsu Province.

Covering an area of 10.72 × 10^4^ km^2^, Jiangsu province is located in the eastern coast region of China at the Yangtze River Delta urban agglomeration, spanning a longitude from 116°18′E to 121°57′E and latitude from 30°45′E to 35°20′E (see Figure 1), with the Yellow Sea on the east side, the Anhui province and the Shandong province on the northwest side, and Zhejiang province and Shanghai on the southeast side. Jiangsu province is one of the most developed regions with one of the highest population densities in China, but with only 1.1% of the total land area in Mainland China, and accounting for 10.4% of the total GDP, ranking second amongst all provinces in 2017 [39]. The permanent population of Jiangsu province has increased from 67,669 thousand to 78,693.4 thousand, of which the urban population increased from approximately 21.6% to 60.6% of the total during the years from 1990 to 2010 [40]. The regional GDP increased from 141.6 billion Chinese Yuan (CNY) to 4142.55 billion CNY during the same research period, reflecting a rapid economic expansion of nearly 30 times. Within this period, the land use situation has experienced a great change related to the rapid urbanization process. For instance, the built-up land expanded from 12,280.82 km^2^ to 20,064.89 km^2^ [41]. There exists an increasing conflict between the land use ecology and the human societal development, which has already resulted in a series of problems like environmental pollution, land degradation, biodiversity loss, etc. [42,43].

The area of study consists of thirteen prefecture cities, which are Nanjing (the capital city), Wuxi, Changzhou, Suzhou, Zhenjiang, Nantong, Yangzhou, Taizhou, Xuzhou, Lianyungang, Huai’an, Yancheng, and Suqian. The whole province is divided into three parts based on the urbanization level and development period: Southern Jiangsu, including Nanjing, Suzhou, Wuxi, Changzhou, and Zhenjiang; Central Jiangsu, including Yangzhou, Nantong, and Taizhou; and Northern Jiangsu, including Huai’an, Xuzhou, Lianyungang, Yancheng, and Suqian [44]. Jiangsu is of an eastern-Asian monsoon climate. The annual temperature is approximately between 13.6 °C to 16.1 °C with efficient rainfall of nearly 1000 mm annually. Owing to mild climate, Jiangsu is called the land of fish and rice, and is one of the most important grain foundations of China [41].

### 2.2. Research Data

● Land Use Data

The land use data used in this paper were initially derived from the historical Landsat thematic mapper satellite imagery, provided by the Yangtze River Delta Science Data Center, National Science and Technology Infrastructure of China, and National Earth System Science Data Sharing Infrastructure (http://nnu.geodata.cn:8008) as in Reference [45]. The available cloud-free Landsat images used were from 1990, 1995, 2000, 2005, and 2010, with an accuracy higher than 94.3%, which was suitable for the large-scale spatial analysis with the precision of a 1:100,000 scale geography map. In this paper, we chose three time phases from 1990, 2000, and 2010 to study the land use evolution trend during this twenty-year period with a rapid urbanization trend.

● Basic Data on the City

This paper used basic urban data for Jiangsu province provided by the Yangtze River Delta Science Data Center, National Science and Technology Infrastructure of China, and National Earth System Science Data Sharing Infrastructure (http://nnu.geodata.cn:8008) [45]. This dataset included the administrative region boundary, capital city, urban center, road network, and location of transportation stations. The data was stored in a shape file format. 

● Socio-Economic Data

The socio-economic data used in this paper was derived from the Statistics Bureau of Jiangsu Province (http://tj.jiangsu.gov.cn/col/col4009/index.html). The dataset provided the information on the socio-economic related issues of 13 cities in Jiangsu province, including the yearly population, urban and rural population, GDP of three industries, employment, retail, people’s living conditions, government finance, resources and environment, energy, investment in fixed assets, agriculture, industry, construction, etc. [39,40,41,46]. The data was stored in an Excel file format. 

● Point of Interest Data

The point of interest data of Jiangsu province was derived from the Baidu map through the web application programming interface (API) provided by the Baidu Company. The data collection and cleaning were done by the Yangtze River Delta Science Data Center [45]. This dataset provided the location information of equipment in the research region, for example the name, longitude, latitude, administrative district, etc. The POI data was divided into six categories and 21 subtypes: the six categories were the residential point, public management and public service function point, commercial service point, industry point, transportation and road point, green area and square point. The data was stored in an Excel file format.

### 2.3. Method

#### 2.3.1. Land Use Types Classification

The original dataset had six categories of land use types, which were arable land, forestry land, grassland, water area, urban and rural built-up land, and unused land. The six categories were further divided into 31 subtypes. For example, the arable land was further divided into dry land and paddy field; the forestry land was further divided into closed forestry land, open forestry land, shrub forestry land, and other types of forestry land. Based on the land use planning of Jiangsu province, considering the land use purpose and management characteristics, we distinguished the main categories of different land use types and determined the similarities. Jiangsu province has a long costal line of over 1000 kilometers, thus the intertidal zone is an important resource for land use storage. We separated the intertidal area out of the water area when doing the analysis (see Table 1).

**Figure 2:**
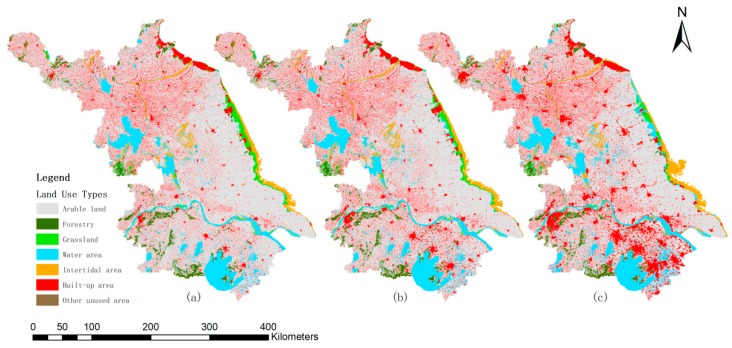
Land use classified map of Jiangsu province from 1990 to 2010: (**a**) Land use situation in 1990; (**b**) Land use situation in 2000; (**c**) Land use situation in 2010.

Based on the classification system above, we generated LUCC situation maps for Jiangsu province over the research period in 1990, 2000, and 2010 (see Figure 2), using the ArcGIS software (ESRI, Redlands, CA, USA) and Python 3.0 (Python Software Foundation, Beaverton, OR, USA). Then, we were able to perform further analyses to calculate the land use degree and land use change dynamic degree of Jiangsu province, and to conclude the driving forces of LUCC and generate relevant thematic maps.

#### 2.3.2. Markov Transition Matrix Model

The Markov transition matrix model is used to describe the change in situation of each land use type between study periods [47,48]. We generated the transition matrix of Jiangsu’s land use from 1990 to 2000 and from 2000 to 2010 to analyze the past changing situations, and also for the simulation of future trends. The calculation formula for the Markov transition matrix model is as follows:

(1) $$S_{ij}=\left[\begin{array}{ccc} S_{11} & \cdots & S_{1n}\\ \vdots & \ddots & \vdots \\ S_{m1} & \cdots & S_{mn} \end{array}\right]$$

where *S* is the area of each type of land use; *i* and *j* are the land use types before and after the research period during the transition; *i*, *j*, *m*, and *n* in this paper represented the land use type code, and were equal to 1, 2, 3, …, 7 as we only had seven types of land use in Jiangsu’s land use classification system. Based on the above calculation, we generated two transition matrixes, and calculated the reduced and increased amount of each land use type.

#### 2.3.3. Land Use Dynamic Degree Model and Land Use Degree Model

To further study the dynamic of land use change situation, we applied the land use dynamic degree model to quantify the dynamics [49,50]. This model described the annual change rate of each type of land use change. The formula is as follows:

(2) $$S=\left\{\overset{n}{\underset{ij}{\sum }}\left(\frac{dS_{i-j}}{S_{i}}\right)\right\}\times \left(1/t\right)\times 100\%$$

where *S* is the land use dynamic degree during the research time *t*; S*_i_* is the total area of land use type *i* in the whole research area; $dS_{i-j}$ is the total area of land transited from type *i* to type *j* during the research time period *t*.

The land use degree is a description of the impact on the environment made by mankind. The natural land is used at different degrees by people in various use types and it is affected by human activity at different degrees, for example, the arable land, built-up land, etc. The unused natural land has a very low effect from mankind while artificial non-reclaimed land has the highest effect. The land use degree model expresses the comprehensive usage situation of land in a certain area and it helps to forecast future usage and changes. To quantify the land use degree, in the Database of Natural Resource of China (DNRC), Liu separated the land use types into four categories (see Table 2) based on the ecology perspective and the effect degree by humans, and gave each type a certain index to further describe the land use degree in a certain research region [37,51,52,53]. The land use degree model formula is as follows:

(3) $$∆I_{b-a}=I_{b}-I_{a}=\left\{\left(\overset{n}{\underset{i=1}{\sum }}A_{i}\times C_{ib}\right)-\left(\overset{n}{\underset{i=1}{\sum }}A_{i}\times C_{ia}\right)\right\}\times 100$$

where $\Delta I_{b-a}$ is the change of land use degree from the research time year *a* to year *b*; *I_a_* and *I_b_* are the land use degree in the research year a and year b; *A_i_* is the land use degree index for land use type *i*, detailed as shown in Table 2; *C_ia_* and *C_ib_* are the percentage of the area of land use type *i* at the time *a* and *b*, respectively. 

#### 2.3.4. Logistic Regression Analysis

The logistic regression model is one of the most popular models to investigate the urban expansion pattern in environmental modeling. The statistical driving factors analysis was first carried out with the support of SPSS Version.23 software (IBM, New York, NY, USA). We built the binary logistic regression model to identify the relationship between multiple variables of driving forces factors and the LUCC [54,55]. 

**Figure A1:**
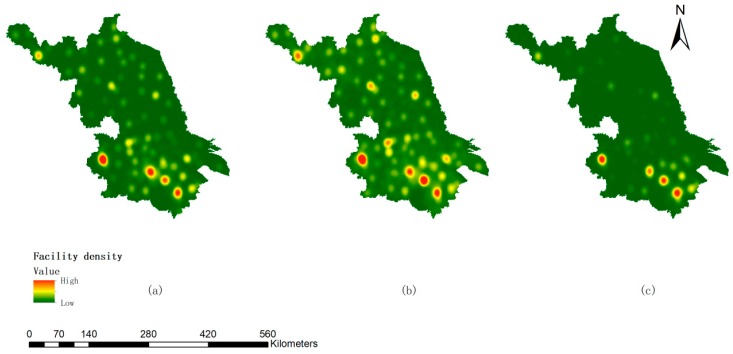
Density of service facility: (**a**) Density of commercial service facility; (**b**) Density of another service facility; (**c**) Density of transportation service facility.

Natural environment, land use management, and socioeconomic human activity have been recognized as the main driving factors of LUCC by the International Geosphere and Biosphere Project (IGBP) and the International Human Dimensions Program (IHDP) [1,5,56]. This comprises, the natural environment which includes the temperature, topography, etc.; the land use management which presents the local government policies, such as comprehensive urban planning and zoning code; and the socioeconomic human activity which includes the population change, education level, social economic structure, economic development, and so on. In theory, for the purpose of learning about the driving factors, all the components should be programmed. However, considering the local situation of Jiangsu province and the data availability, the major driving forces were identified. In this study, the major factors were grouped into three subsystems: (1) Natural environment factors, including the elevation, slope, and distance to the water area, which may affect the efficiency of arable land, intertidal area conversion, and the selection of the urban area. (2) Land use management factors, including the distance to highway, distance to urban road, distance to urban center, the density of urban commercial service facility, the density of the transportation facilities, and density of other service facilities (Figure A1). (3) Socioeconomic human activity factors, including the gross domestic product (GDP), population change, per capita household income, and per capita housing area. Social economic development may require more urban area to pursue development, while human activity may change the function of land in the corresponding area. The values of the above factors were obtained from the land use information, planning maps, Jiangsu Statistics Yearbooks, and POI data. 

For the purpose of examining the relationships between LUCC and the above driving factors, the logistic regression model was applied. A set of potential driving factors were identified from the understanding of LUCC determinants. The simulated driving factors with an insignificant contribution to the LUCC were eliminated and the significant factors were retained during the model testing process. The logistic regression equations were expressed as follows:

(4) $$P_{\left(y=\frac{m}{x}\right)}=\frac{\exp\left(\beta_{0m}+\beta_{1m}x_{1}+\beta_{2m}x_{2}+\ldots +\beta_{km}x_{k}\right)}{1+\sum_{i=2}^{e}\exp(\beta_{0i}+\beta_{1i}x_{1}+\beta_{2i}x_{2}\ldots +\beta_{ki}x_{k})}$$

(5) $$\ln⎣\frac{p\left(y=\frac{m}{x}\right)}{p\left(y=\frac{1}{x}\right)}⎦=\beta_{0m}+\beta_{1m}x_{1}+\beta_{2m}x_{2}+\ldots +\beta_{km}x_{k}$$

where *p* is the occurrence probability of a certain land use type change; *y* is the reference variable; *x* is the potential driving factors; *p*(*y*=1) refers to the probability that the land use type remains unchanged; *p*(*y*=*m*/*x*) is the probability that the land use type changes into type m; $\frac{p\left(y=\frac{m}{x}\right)}{p\left(y=\frac{1}{x}\right)}$ is the odds ratio of a group of events; $\beta_{0}$ is the constant; $\beta_{n}$ is the coefficient of the *n*th driving factor. 

The odds ratio was calculated to express the constant effect of a predictor. The Wald value test was used in this model to explain whether this variable had a correlation with the LUCC. Non-parametric receiver operating characteristic (ROC) curve tests were applied to confirm the correlation model adjustment. A null value meant that this statistical test had a *p* value lower than 0.05.

## 3. Results

### 3.1. Spatial and Temporal Patterns of Land Use and Cover Change

**Figure 3:**
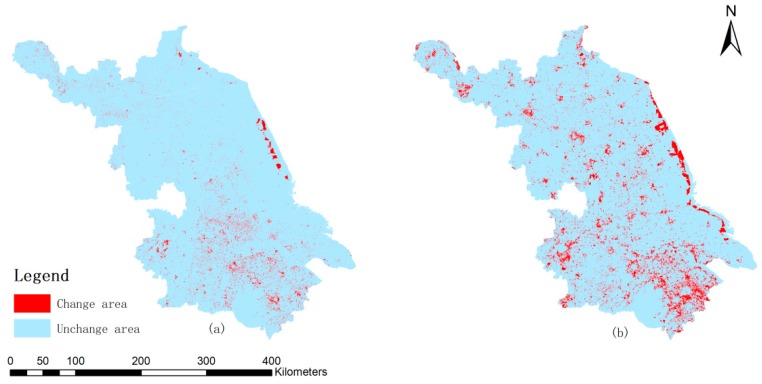
Distribution map of change situation of land use type of Jiangsu province from 1990 to 2010: (**a**) Land use type change situation from 1990 to 2000; (**b**) Land use type change situation from 2000 to 2010.

Spatial superposition of land use data was used to study the spatial and temporal characteristics of LUCC in Jiangsu province over the past twenty years. With the support of the Arc/Info Grid software module (ESRI, Redlands, CA, USA), we compared each two sections of land use change situations and generated the spatial characteristic maps from 1990 to 2010 (see Figure 3). The most significant land use change was caused by the urbanization development. It was clear that the southern Jiangsu region developed first and then came the central and northern Jiangsu regions. From 1990 to 2000, the changes of land use types were mainly distributed in the southern Jiangsu region, as the southern region had a higher urbanization level at that time. The intertidal area along the eastern costal region expanded as a result of the deposits of sediment at the Yellow Sea. From 2000 to 2010, there were more obvious changes all across the province’s regions compared to that of 1990 to 2000, while the southern region had a slightly higher density than the central and northern Jiangsu regions. The main changes in this period were the continuous expansion of urban built-up land and the greater reduction in arable land. The eastern costal region had even more obvious changes than before, as some of the intertidal area changed into arable land or forestry land and the sea continuously deposited sediments. 

**Figure 4:**
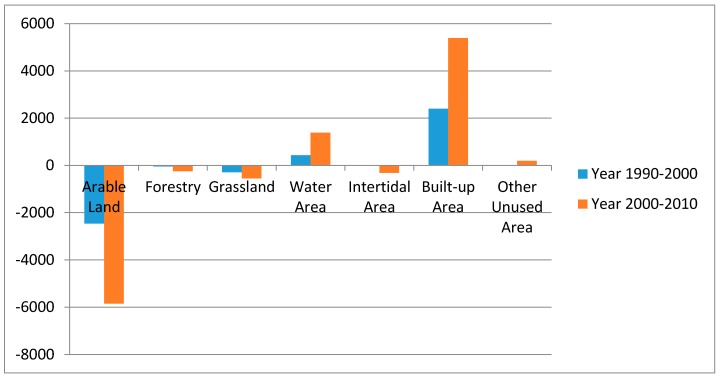
Statistical amount changes of land use in Jiangsu province (unit: km^2^).

With a further analysis of the amount of each type of land use, we generated a statistical amount changes map for the study period (see Figure 4). It was clear to see that the total amount of arable, forestry land, grassland, and intertidal area was continuously decreasing, while the water area and built-up area had an increasing trend from 1990 to 2010. To be specific, from 1990 to 2000, the reduction of arable land and grassland was nearly equal to the increase of built-up area and water area, while the changes of forestry, intertidal area, and other unused areas were much less. From 2000 to 2010, there was a greater amount in the reduction of arable land, forestry, grassland, and intertidal area, as well as a greater amount of the increase of water area and built-up area.

From the attribute calculation of each land use type, we conducted a further comparison of LUCC during the two research phases. The most obvious change was the recession of arable land, where it covered 69.88% of the total area at an amount of 72,296.03 km^2^ in 1990, but was reduced to 67.49% by a reduction amount of 2461.84 km^2^ in 2000, and it continued to decline to 61.83% by an area of 5850.57 km^2^ to a new total area of 63,983.93 km^2^. Conversely, the second significant change was the increase in the built-up area, wherein it covered 11.87% of the total area at an amount of 12,280.82 km^2^ in Jiangsu province in 1990, which changed to 14.18% through an increase of 2394.39 km^2^ in 2000, and then continuously increased to 19.39% by an area of 5389.68 km^2^ in 2010 to a new total area of 20,064.89 km^2^. 

The above mentioned data gave us a clear insight into the main characteristics of land use and cover change in Jiangsu province from 1990 to 2010. There was an obvious increase in the built-up area as Jiangsu province was experiencing a rapid urbanization period. There was an overall decrease in arable land which was taken over for urban expansion, though the arable area still accounted for the major land use type as Jiangsu province is a big agricultural province. The water area and other unused area increased significantly, while the forestry, grassland, and intertidal area decreased rapidly mainly caused by natural or other causes.

### 3.2. Land Use Transition Matrix from 1990 to 2010

We used the Markov transition matrix model to calculate the land use situation transition matrix in order to analyze the change direction of each type of land use. We employed the tabulate command of the ArcGIS software to calculate the transition matrix of LUCC in Jiangsu from 1990 to 2000 and then from 2000 to 2010 (See Table 3 and Table 4). Over 20 years, there was a steady reduction of arable land changing into other types of land use, while the most significant change type was the transition into built-up area. From 1990 to 2000, the recession amount of arable land was 2621.59 km^2^, out of which 86.92% changed into the built-up area. From 2000 to 2010, the recession amount of arable land was 6491.61 km^2^, out of which 78.19% changed into the built-up area. It showed that the major transition pattern of Jiangsu province during 1990 and 2010 was the transition between the arable land and built-up area due to the urban expansion and industrial development. Owing to the environmental protection policy of returning arable land back to the natural use, there was also some arable land changed into the water, intertidal, forestry, and grassland areas, as well as conversions into arable land. To be specific, from 1990 to 2000, 13.08% of the total changed arable land was changed into forestry, grassland, water, and intertidal areas, out of which the water area accounted for the largest share with 332.11 km^2^. From 2000 to 2010, this trend continued with 15.89% of total transition arable land changing into forestry, grassland, water, and intertidal areas, where water also accounted for the largest share with even more area of 885.36 km^2^. The intertidal and water areas transitioned into each other at different locations. Moreover, there was obvious grassland that changed into water areas at an area of 101.6 km^2^ from 1990 to 2000, and then 283.64 km^2^ from 2000 to 2010. Moreover, there was some grassland that changed into intertidal area from 2000 to 2010 at an area of 79.02 km^2^. The above water ecology region transition was mainly caused by construction of ecology parks and natural preservation parks along the eastern seaboard region. 

### 3.3. Land Use Dynamic Degree and Land Use Degree

The Land Use Dynamic Degree Model and the Land Use Degree Model are reflections of the LUCC evolution process and show the future change trend. We employed the land use dynamic degree model described in Section 2.3 to express the land use dynamic variation, and the change area within the research period (see Table 5). 

It was clear to see that from 1990 to 2000, the most obvious land use transition dynamic degree was the built-up area at 19.5%, and it showed a positive trend. Then the grassland was also significant showing -16.04% in a negative decreased development direction, whilst for the rest, the land use dynamic degree of the water area (4%), other unused area (−4.13%), and arable land (−3.41%) were also obvious. From 2000 to 2010, the other unused areas increased by an amount of 196.27 km^2^ though it had the most obvious dynamic degree of 995.79%, because the base amount was pretty small at only 19.71 km^2^. Next was the built-up area of 36.73%, whilst the dynamic degrees of the rest of the land use types were all active. Comparing the land use dynamic degree of two study periods, the built-up area’s dynamic degree from 2000 to 2010 was 1.88 times than that from 1990 to 2000. As a whole, the overall land use dynamic degree of Jiangsu province from 2000 to 2010 was 2.48 times much more than that from 1990 to 2000, with a positive development trend. During the 20 year research period, the LUCC in Jiangsu region was active and had a pronounced changing trend. 

In order to further the analysis, the land use situation from the human ecology effect was examined, where we employed the land use degree model to describe the index of each land use type within the whole Jiangsu province (Table 6). When the value of the land use degree was above zero, this type of land use was experiencing a developing period, while on the contrary, it was experiencing a recession period. The model results dataset suggested that there was an overall increasing trend of land use degree from 1990 to 2010, as Jiangsu province is located in the Yangtze River Delta developed region that is experiencing an accelerating economic development. Under this rapid development background, the urbanization trend accelerated with urban development, thus, human activity has had a relatively larger effect on the ecology, which is reflected as LUCC. 

The built-up area, water, and other unused areas were experiencing an increasing period at different degrees. It showed that the urban expansion in Jiangsu province was undergoing successful development, as the land use degree of the built-up area was much larger than the others; while the water and other unused areas achieved successful development due to the environmental protection policy of returning arable land back to its natural use. The arable land, forest, grassland, and intertidal areas were all experiencing a recessionary period over these 20 years, reflecting that the high urbanization trend absorbed a large amount of non-built-up land to be changed into urban built-up area. The land use degree of the whole Jiangsu province showed an increasingly active trend, as the land use degree from 2000 to 2010 was more than twice that from 1990 to 2010. This may cause the other unused and intertidal areas to be converted into arable land or changed into grassland and forestry, while the grassland and forestry can be reclaimed into arable land, and the arable land and other types of land changed into built-up areas, etc. Some areas may be different due to local land use policy with different development periods within the whole province. 

### 3.4. Logistic Regression Analysis

The logistic regression model was applied to analyze the driving factors of the LUCC. A total of 100,000 random points in Jiangsu province were created using the “Create Random Points” tool in ArcGIS 10.3. We then used the tool “Extract Value to Points” to apply each spatial information to the corresponding random points. To analyze the natural and socio-economic driving factors on the LUCC, we employed the statistical data from the data center, and generated the social related variables based on the newly released point of interest data. We generated the elevation, slope of Jiangsu province, GDP, population change, per capita household income, and per capita housing area directly from the statistical data; calculated the distance to the water area, the distance to the highway, distance to the urban road, and distance to the urban center based on the city basic data; and generated the density of commercial service facility, density of transportation facility, and density of other service facility. To confirm the accuracy of the regression result, we used the non-parametric receiver operating characteristic (ROC) curves, and the result showed that all the land use types had a ROC test value that was higher than 0.7.

The odds ratio (see Table 7) explained the relationship between the variables and the changes of each type of land use. The research result suggested that the thirteen factors forced the LUCC of each type at different directions. Amongst these factors, the elevation only had a relation with the arable land transition, grassland conversion, and the water area loss; the slope affected the location of arable land positively and that of forestry and intertidal area negatively. The distance to water area and the distance to highway affected the changes to all types of natural land, as the water resource is an important consideration in planning site selection. The distance to the urban road affected the grassland positively. The distance to the urban center had a positive effect on the forestry, grassland, and water, while it had a negative effect on built-up area. The density of the transportation facility, the GDP, population change, and per capita housing area all affected the arable land, while the urban construction and economic development consumed a large amount of arable land. These socio-economic related factors all had a positive effect on the built-up area. More details on the driving forces are discussed in Section 4.

## 4. Discussion

The results from the statistical models above showed a detailed analysis of the specific spatio-temporal patterns of LUCC in Jiangsu province over the 20 year rapid development period from 1990 to 2010. The statistical analysis in Section 3.4 described some quantifiable relationships between LUCC and certain driving forces. Natural issues like elevation had a relatively low effect as Jiangsu province is located in the middle and lower regions of the Yangtze River Plain. The construction related reasons may have had more of an effect on the changes to the urban region, as Jiangsu province has experienced a rapid urbanization process over the research period. In general, LUCC was influenced by the natural environment, government policy which is hard to quantify, social economic development, and social technology development. Over the study period, Jiangsu province had a stable economic and social development, where strong economic and population growth were the main driving forces of LUCC within this 20-year small scale time period. 

### 4.1. Driving Forces of Human Activity

**Figure 5:**
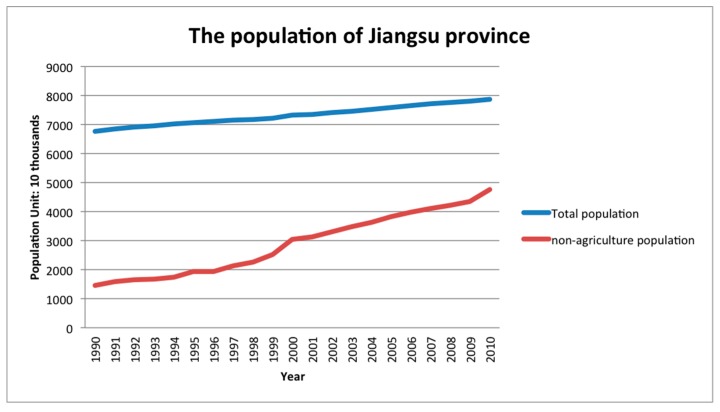
Population changes in Jiangsu province (unit: 10 thousand).

The land use situation has been influenced by humans through a series of socioeconomic activities over a long-term period [2,57]. Li pointed out that the driving factors of LUCC should be explained by the land use situation change, activity by land users, and the social land use management [2]. As a whole, LUCC is a result of the combination of natural and socio-economic change. Human activity related driving forces of LUCC are mainly population increase, urban expansion, urban construction, human migration, and some other government policy issues. Based on Jiangsu province Statistical Yearbook [40], the permanent population of Jiangsu province, the percentage of the population increase, and the percentage of people working for non-agriculture sectors are shown below in Table 8 and Figure 5. It is clear that during this 20-year study period, Jiangsu province had a stable increase in the total permanent population with a linear development trend, and it showed a continued upward trend in the near future. It had a negative effect on the arable land while positively affecting the built-up area expansion. However, a balance should be struck between the conversion of arable land and the built-up area in order to achieve and maintain sustainable development and to reduce the risk to food security as Jiangsu province is an agriculture oriented province in China with a large population [10,58]. The percentage of the population working for non-agriculture is continuously increasing at an accelerating rate above the national reported average level, and it suggests a continued trend. The rapid population increase and urbanization situation of the urban area of Jiangsu province have resulted in housing price rises, public transportation congestion, environmental pollution, rising basic living costs, and food security issues, which further accelerate the conversion from arable land to built-up area. Thus, some people have begun to move to sub-urban areas to attain affordable living, such as Jurong which is near Nanjing, Kunshan which is near Shanghai, Pukou New District, etc. This behavior promotes the urbanization process further to the nearby less developed regions, and without a doubt it will continue to accelerate the whole urbanization process of Jiangsu province, whilst having a direct effect on the land use type changes of the built-up area expansion.

With the remarkable change in people’s living standards, residents have begun to seek a better living environment as one of the main driving forces of land use type changes. According to Jiangsu province Statistical Yearbook [40], the per capita housing area was 9.06 m^2^ per person in 1990, increasing to 25.54 m^2^ per person in 2000, and then continuing to increase to 33.39 m^2^ per person in 2010. At the same time, the real estate market has developed at an unbelievable pace. While the GDP of the real estate industry was 2.702 billion Chinese yuan (CNY) in 1990, it increased to 29.82 billion CNY in 2000, and 260.1 billion CNY in 2010. The GDP of the real estate sector developed rapidly with a factor of 96.3 times over the 20-year period. This rising demand for residential areas will lead to the expansion of the built-up area and may occupy more natural land, promoting the transition between arable land, forestry, grassland, or water areas into built-up area to meet the urban expansion demand. Conversely, the government has set up a series of land use protection plans, such as returning forestry to arable land, and thousands of clear farmland projects, with the purpose of protecting the land use red line [59]. This contributes to reducing the land use pressure to a certain degree. During the study period, there was a total of 1295.7 km^2^ of arable land converted into other types of natural land, which mostly became commercial forest and culture water [60]. These converted areas can not only provide an economic foundation for local farmers, but they also promote environmental protection.

Since the economic reform in 1978, there was significant progress in global communication and technology development. As part of modern societal development, Jiangsu province has built nine airports, and an efficient highway network, which are usually located outside the urban center. Moreover, there have been an increasing number of mega events and international conferences being held in Jiangsu province, which led to the construction of an event center and a nearby hotel, and may cause a certain effect on the land use changes. For example, Nanjing held the Youth Olympic Games in 2014, resulting in a lot of construction programs that occupied a certain amount of arable land in the Hexi district to build an Olympic Village. This kind of activity accelerates the urban infrastructure investment over a short period, increases the public service level, and promotes LUCC without doubt [61]. 

### 4.2. Driving Forces of Socio-Economic Development

Socio-economic development is usually regarded as one of the most important driving forces of LUCC during the rapid economic growth period. In this research period from 1990 to 2010, Jiangsu province experienced accelerating economic development. The direct reflection is the gross domestic product (GDP) increase, which was 36.87 billion Chinese yuan (CNY) in 1990, and grew to 306.95 CNY in 2000, and continued to increase to 1713.14 CNY in 2010. The total GDP increased by a factor of 46.46 times over the 20-year research period. Additionally, the per capita GDP grew rapidly, where it was 2109 CNY in 1990, increased to 11,765 CNY in 2000, and further increased to 52,840 CNY in 2010. However the growth rate of per capita GDP at a factor of 25 times was less than half that of the total GDP, which was the result of a simultaneous population growth. This overall economic development brought new demand for residential living environments, which may have led to the changes in land use types. 

**Figure 6:**
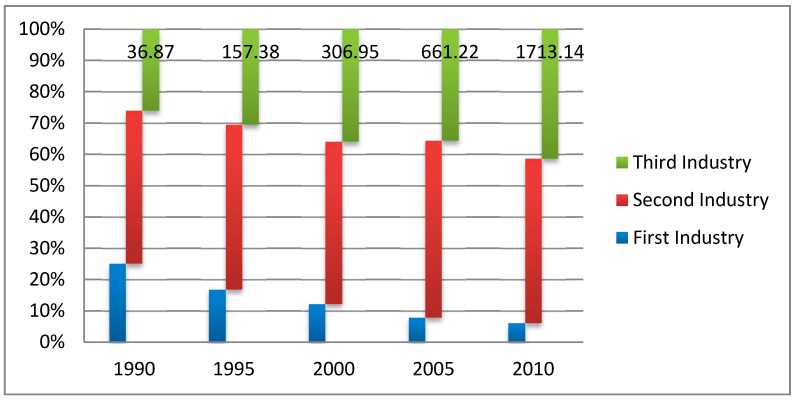
Industrial structure of Jiangsu province expressed by gross domestic product from 1990 to 2010.

To further study the socio-economic development, the industrial structure also underwent a gradual transformation (Figure 6). We could observe that the industrial structure optimization of the primary industry experienced an accelerating decrease, while the second industry remained slightly stable, and the tertiary industry kept increasing. To be specific, the proportions of the three industries were 25.08%, 48.9%, and 26.03% in 1990; changed to 12.26%, 51.86%, and 35.89% in 2000; and then optimized into 6.13%, 52.51%, and 41.35% in 2010. The tertiary industry was directly reflected by the kinds of service facilities in the research area, which were analyzed in Section 3.4 and showed a clear relation with the LUCC. The industrial structure changed faster than the land use dynamic degree. In Jiangsu province, an agriculture oriented province in eastern China, its industrial structure decides the urban function structure change and the corresponding population transition between the agriculture population and non-agriculture population, which was an important pattern during the rapid urbanization process. As one of the significant driving forces behind LUCC, the planning department should pay attention to the inner optimization of the primary industry, limit the expansion of the second industry, and increase the tertiary industry appropriately. 

## 5. Conclusions

In this study, we analyzed the spatio-temporal patterns of LUCC over a rapid development period, taking a case study of Jiangsu province from 1990 to 2010. We analyzed the land use type, land use distribution, land use transition matrix between research time periods, land use dynamic degree, land use degree, and the potential driving forces. From the above analysis, the LUCC in Jiangsu can be characterized by three major trends: First is the substantial conversion of agricultural land into non-agricultural land from 1990 to 2010, for example, the rapid expansion of the built-up area, the obvious recession of arable land, and the increase of natural areas for protection purposes. Second, both the land use dynamic degree and land use degree were increasing. Third, the conversion of arable land is a serious issue which deserves more attention to ensure food security, as Jiangsu is an agriculturally-oriented province. 

In this research, we used remote sensing technology, the ArcGIS software, new-type POI data, and some statistical methods to analyze the evolution process of the LUCC to obtain an accurate result, and determined the driving forces, as well as providing suggestions for the optimization of future land use planning. In order to achieve a balance between human–land interactions, and to learn more about the future simulation of land use to support decision making in the urban planning of Jiangsu province, the land use dynamic degree, land use degree, and driving factors were proposed to analyze the spatio-temporal evolution of the LUCC at a regional scale. This dynamic change showed a rapid spread of urban construction land, especially around the city centers to the surrounding areas, while the human activities in the urban area had a relatively higher expansion trend. In terms of the land use structure conversion over years, a large amount of arable land was converted into built-up land within this study period. Moreover, there was a significant transition from forestry, grassland, and water areas to built-up areas due to the rapid urban sprawl, as well as the remarkable progress of people’s living standards. To boost the sustainable usage of the very limited land resources in developed regions, it is highly necessary for planning departments to seek a win–win method in the dilemma of economic development and arable protection, and to optimize land use planning using various methods, such as adopting satellite towns to replace disorderly central urban expansion. The results found in this study can also be applied to similar regions experiencing rapid economic development. In the future, we should focus more on highly-efficient land use as mega city centers are facing the problem of land use pressure, and we should also pay attention to the sustainable development of land use and global environmental change. 

## Figures and Tables

**Table 1:** The classification system of land use types of Jiangsu province.

Code	Name	Explanation
1	Arable Land	Land for growing the crops, including the dry land, paddy field, and the intertidal area covered with planted crops more than three years
2	Forestry	Land for planting trees, including closed forestry land, open forestry land, shrub forestry land, and other types of forestry
3	Grassland	Land growing grasses, sedge, and shrubs covering more than 5% of total area
4	Water Area	Natural area covered with water or the place with water conservancy facilities
5	Intertidal Area	Land exposed to water that has not been used for many years
6	Built-up Area	Urban and rural built up area, including residential area, transportation area, public building area, industry area, commercial area, etc.
7	Other Unused Area	Other kinds of land that have not been used for years

**Table 2:** Classification and index of land use types.

Classification Type	Natural Unused	Natural Reclaimed	Artificial Reclaimed	Artificial Non-Reclaimed
Land use type	Other unused area	Forest, grassland, water area, and intertidal area	Arable land	Built-up area
Index of land use categories	1	2	3	4

**Table 3:** Land use situation transition matrix in Jiangsu province from 1990 to 2000 (km^2^).

1990/2000	Arable Land	Forestry	Grassland	Water Area	Intertidal Area	Built-up Area	Other Unused Area
Arable Land	69,674.45	3.73	0.38	332.11	6.58	2278.79	0.00
Forestry	3.24	3376.05	0.1	2.21	0.00	41.83	0.42
Grassland	109.02	0.00	1486.16	101.5	0.00	78.85	0.00
Water Area	16.05	0.6	3.64	10,672.69	12.82	10.22	0.00
Intertidal Area	0.36	0.00	0.00	30.78	2915.46	1.52	0.00
Built-up Area	15.68	0.00	0.32	1.02	0.00	12,263.8	0.00
Other Unused Area	0.00	0.00	0.00	1.28	0.00	0.00	19.28

**Table 4:** Land use situation transition matrix in Jiangsu province from 2000 to 2010 (km^2^).

2000/2010	Arable Land	Forestry	Grassland	Water Area	Intertidal Area	Built-up Area	Other Unused Area
Arable Land	63,342.59	64.77	9.35	885.36	8.12	5459.98	64.03
Forestry	176.49	3047.52	0.01	7.33	0.28	112.17	34.26
Grassland	121.05	1.53	815.48	283.64	79.02	125.01	64.95
Water Area	85.57	1.2	2.52	10,792.73	41.6	220.45	0.64
Intertidal Area	36.45	0.23	77.91	292.65	2486.31	39.55	0.24
Built-up Area	221.49	11.05	31.17	266.53	2.04	14,106.06	36.87
Other Unused Area	0.29	1.09	0.00	1.54	0.13	1.67	14.99

**Table 5:** The land use dynamic degree of Jiangsu province from 1990 to 2010.

Land Use Type	From Year 1990 to 2000		From Year 2000 to 2010	
	Change Area (km^2^)	Land Use Dynamic Degree (%)	Change Area (km^2^)	Land Use Dynamic Degree (%)
Arable land	−2461.84	−3.41	−5850.27	−8.38
Forest	−45.79	−1.34	−250.67	−7.42
Grassland	−284.85	−16.04	−554.24	−37.18
Water area	428.69	4.00	1385.07	12.43
Intertidal area	−14.78	−0.50	−315.84	−10.77
Built-up area	2394.39	19.50	5389.68	36.73
Other unused area	−0.85	−4.13	196.27	995.79
Sum of changes	5631.19	5.44	13,942.04	13.47

**Table 6:** Land use degree of Jiangsu province.

Land Use Type	From Year 1990 to 2000	From Year 2000 to 2010
Arable land	−7.14	−16.96
Forest	−0.09	−0.48
Grassland	−0.55	−1.07
Water area	0.83	2.68
Intertidal area	−0.03	−0.61
Built-up area	9.26	20.83
Other unused area	0.00	0.19
Sum of total research region	2.28	4.57

**Table 7:** Odds ratio of the driving forces of each land use type based on the logistic regression analysis.

Driving Forces	Arable Land	Forestry	Grassland	Water Area	Built-Up Area	Intertidal Area
elevation	0.889	-	1.014	0.926	-	-
slope	1.003	0.998	-	-	-	0.982
Distance to water area	1.004	0.890	-	0.935	-	0.999
Distance to highway	0.916	0.788	-	-	0.997	1.218
Distance to urban road	-	-	1.014	-	-	-
Distance to urban center	-	1.012	1.000	1.000	0.892	-
Density of commercial service facility	-	-	-	-	1.301	0.793
Density of transportation facility	1.012	-	1.001	-	-	1.001
Density of other service facility	-	1.012	-	-	-	-
GDP	0.722	0.972	-	-	1.163	-
Population change	0.942	-	-	-	1.062	-
Per capita household income	-	0.927	-	-	1.019	-
Per capita housing area	0.602	0.918	-	-	1.179	-

“-” means the result cannot pass the significance test.

**Table 8:** Population of Jiangsu province.

Year	Permanent Population. Unit: Ten Thousand	Percentage of Population Increase	Percentage of Non-Agriculture Population
1990	6766.9	/	21.56%
1995	7066.02	4.42%	27.30%
2000	7327.24	3.7%	41.50%
2005	7588.24	3.56%	50.50%
2010	7869.34	3.7%	47.88%
